# Detection of *Borrelia burgdorferi* s.l., *Anaplasma phagocytophilum* and *Babesia* spp. in *Dermacentor reticulatus* ticks found within the city of Białystok, Poland—first data

**DOI:** 10.1007/s10493-021-00655-x

**Published:** 2021-09-15

**Authors:** Anna Grochowska, Justyna Dunaj, Sławomir Pancewicz, Piotr Czupryna, Piotr Majewski, Mulugeta Wondim, Elżbieta Tryniszewska, Anna Moniuszko-Malinowska

**Affiliations:** 1 Department of Infectious Diseases and Neuroinfections, Medical University of Białystok, Żurawia 14, 15-540 Białystok, Poland; 2 Department of Microbiological Diagnostics and Infectious Immunology, Medical University of Białystok, Waszyngtona 15A, 15-269 Białystok, Poland

**Keywords:** Urban, *Babesia canis*, *Babesia vogeli*, *Babesia venatorum*, *Babesia microti*

## Abstract

Pathogens carried by ticks pose a threat to both human and animal health across the world. Typically associated with rural landscapes, ticks appear to adapt well to life in urban recreational areas. Although *Dermacentor reticulatus* is commonly found across Europe, data on the prevalence of pathogens in this tick species, in an urban environment, are very limited. PCR was used to examine 368 *D. reticulatus* individuals collected in the Zwierzyniecki Forest Nature Reserve in Białystok, Poland. In total, 10.3% of ticks were infected, with *Babesia* spp. (9.2%), *Anaplasma phagocytophilum* (0.8%) and *Borrelia burgdorferi* sensu lato (0.3%). *Rickettsia* spp., *Bartonella* spp., and *Coxiella burnetii* were not detected. Sequence analysis for *Babesia*-positive samples identified 79.4% of them as *Babesia canis*, 8.8% as *Babesia microti*, 5.9% as *Babesia* spp., 2.9% as *Babesia venatorum*, and 2.9% as *Babesia vogeli*. Results obtained in this study indicate that *D. reticulatus* ticks found within the urban premises of the study area are infected with at least three pathogens and therefore are an important factor in public health risk for tick-borne diseases.

**Supplementary Information:**

The online version contains supplementary material available at 10.1007/s10493-021-00655-x.

## Introduction

After *Ixodes ricinus*, *Dermacentor reticulatus* is the second most common tick species encountered in Europe (Didyk et al. 2017; Roczeń-Karczmarz et al. 2018; Stańczak et al. 2018)*.* This species acts as a vector for multiple viral, bacterial and protozoan pathogens (Didyk et al. 2017), such as *Babesia* spp., Tick-borne encephalitis virus (TBEV), *Borrelia burgdorferi* sensu lato, *Anaplasma phagocytophilum*, *Rickettsia* spp., *Bartonella* spp., *Coxiella burnetii*, *Francisella tularensis* and *Francisella*-like endosymbionts (Mierzejewska et al. 2015; Zając et al. 2017; Roczeń-Karczmarz et al. 2018).

Larvae and nymphs of *D. reticulatus* parasitize exclusively on various rodents, whereas adults feed on larger mammals, such as deer, horses, dogs or wild boars. Although *D. reticulatus* ticks rarely feed on humans (Dautel et al. 2006; Mierzejewska et al. 2015), they are still an important part of the pathogen circulation in the environment and therefore their infection rates should be investigated. Public health risk posed by *D. reticulatus* is significant, especially because of their exemplary abilities to survive and adapt to new environments, as well as their high reproduction rate. A fertilized *D. reticulatus* female lays over 7000 eggs, out of which a large number of larvae may survive (Šimo et al. 2004). Furthermore, in comparison to *I. ricinus*, *D. reticulatus* develops into subsequent stages at a faster rate and has greater temperature tolerance. Adult *D. reticulatus* ticks can survive for up to 4 years without a blood meal and because they feed on a wide variety of hosts, including wild and domestic mammals, they may spread over large distances while attached to them (Földvári et al. 2016).

Due to the rapidly progressing process of urbanization across the world, an increasing number of landscapes is being transformed into urban spaces. Although ticks are typically associated with rural surroundings, such as forests and meadows, it is now not uncommon to encounter them in parks, cemeteries, playgrounds or other urban green areas (Rizzoli et al. 2014; Akimov and Nebogatkin 2016). It is worth noting that although literature data regarding ticks in cities are comprehensive when it comes to *I. ricinus*, information on *D. reticulatus* in an urban environment is very scarce.

In this study, *D. reticulatus* ticks collected in Białystok, Poland, were tested for the presence of six tick-borne pathogens: *B. burgdorferi* s.l., *A. phagocytophilum*, *Babesia* spp., *Rickettsia* spp., *C. burnetii* and *Bartonella* spp.

## Materials and methods

Questing *D. reticulatus* ticks were collected from the Zwierzyniecki Forest Nature Reserve in Białystok, Poland (53°6′45″N, 23°9′41″E). The area is dominated by hornbeam, oak, pine and birch trees. It is located approximately 2 km from the city center and is popular among residents for recreational activities, such as hiking, biking, dog walking and jogging, among others.

The collection of ticks took place between April and October 2018. Questing ticks were sampled using the flagging method and subsequently placed separately in Eppendorf tubes. Each tick was identified to species and stage using taxonomic keys (Nowak-Chmura 2013) and stored at +4 °C until further DNA extraction.

### DNA isolation

Each tick was crushed individually in a mortar with addition of 1.5 ml of PBS (without Ca^2+^ and Mg^2+^ ions). Homogenate was centrifuged and 300 µl of obtained supernatant was used for DNA extraction. This process was performed with spin column kits (EurX DNA Isolation Kit, Poland) in accordance with manufacturer’s instructions. Afterwards, 100 µl of obtained DNA extracts was stored at −20 °C until further analyses.

### PCR amplification

All PCR reactions were performed on the LabCycler (SensoQuest, Germany). Specimens were tested in pools, each containing five DNA extracts (15 µl of each). Afterwards, if a pool was positive, all its components were examined individually in order to establish the exact number of infected ticks.

#### *Borrelia burgdorferi* s.l. PCR

*Borrelia burgdorferi* s.l. PCR was performed with the *B. burgdorferi* PCR kit (GeneProof, Czech Republic) for in vitro diagnostics. A 120-bp fragment of the 16S rRNA gene encoding small ribosomal subunit was amplified. Final reaction mix volume of 40 µl comprised of 30 µl of MasterMix and 10 µl of the template DNA extract. For minimization of risk of non-specific reactions and maximization of the sensitivity of procedure, ‘hot start’ technology was used. PCR inhibition was controlled by an internal standard in the reaction mix. Possible contamination during preparation was avoided by adding Uracil-DNA-glycosylase (UDG).

Reaction program was designed in compatibility with GeneProof instruction with own modifications and consisted of the following steps: UDG decontamination at 37 °C for 2 min, initial denaturation at 95 °C for 10 min, amplification for 45 cycles (denaturation at 95 °C for 5 s, annealing at 60 °C for 40 s, extension at 72 °C for 20 s) and final extension at 72 °C for 2 min.

#### *Anaplasma phagocytophilum* PCR

To detect *A. phagocytophilum* DNA, a nested PCR was used targeting a fragment of 16S rDNA gene encoding small ribosomal 16S RNA subunit. Reactions were performed with the *Anaplasma* PCR kit (Blirt-DNA Gdańsk, Poland), according to the manufacturer’s instructions. In the first stage (PCR-OUT), the reaction mix volume of 50 μl was obtained by mixing 2 μl of template DNA isolate, 42 μl of PCR-OUT MasterMix, 5 μl of dNTPs and 1 μl of Taq nova polymerase. First course of amplification proceeded the following PCR program: initial denaturation at 95 °C for 2 min, 40 cycles (denaturation at 94 °C for 30 s, annealing at 55 °C for 30 s, extension at 72 °C for 60 s) and final extension at 72 °C for 5 min. In the second stage (PCR-IN), 2 μl of PCR product from the first reaction was mixed with 42 μl of PCR-IN MasterMix, 5 μl of dNTPs and 1 μl of Taq nova polymerase. The course of amplification used in this stage followed the same steps as in PCR-OUT, but in 30 cycles.

#### *Babesia* spp. PCR

For detection of *Babesia* spp., a fragment of 18S rDNA gene was used, encoding a small ribosomal subunit, localized on conservative region V4. All steps for this reaction were constructed experimentally based on previous methods (Piccolin et al. 2006; Pichon et al. 2006). PCR was performed with Taq PCR Core Kit (Qiagen, Germany) with the use of a pair of highly specific primers: 18S rDNA BAB-F2 sense 5ʹ–GAC ACA GGG AGG TAG TGA CAA G–3ʹ and 18S rDNA BAB-R2 antisense 5ʹ–CTA AGA ATT TCA CCT CTG ACA GT–3ʹ (Sigma–Aldrich, Germany) (Pichon et al. 2006; Katargina et al. 2011; Moniuszko-Malinowska et al. 2016; Dunaj et al. 2018).

The reaction mixture (25 µl) contained 2.5 µl of extracted DNA, 5 µl of buffer × 10 with 15 mM MgCl_2_, 2 µl of 25 mM MgCl_2_, 1 µl of 10 mM dNTPs, 1 µl of 20 µM primer sequencing (18S rDNA BAB-F2 and 18S rDNA BAB-R2) and 0.25 µl (5 U/µl) of thermostable Taq DNA polymerase. Amplification took place in the following steps: initial denaturation at 94 °C for 3 min, 40 cycles (denaturation at 94 °C for 40 s, annealing at 58 °C for 60 s, extension at 72 °C for 60 s) and final extension at 72 °C for 10 min.

#### *Bartonella* spp., *C. burnetii* and *Rickettsia* spp. PCR

Diagnostic The Hum PCR BARTONELLA, The Hum PCR Coxiella burnetii and the Vet PCR RICKETTSIA detection kit (BioIngenTech, Chile) were used to detect *Bartonella* spp. *C. burnetii* and *Rickettsia* spp., respectively. Reactions were performed according to the manufacturer’s instructions. Reaction mixture (10.7 µl) contained 2.7 µl of HumPCR *Bartonella* Premixture, HumPCR *C. burnetii* Premixture or VetPCR *Rickettsia* Premixture, accordingly, 6 µl of free water and 2 µl of either sample DNA, negative control or positive control. Additionally, internal control samples were prepared by mixing 2.7 µl of Internal Control Mixture, 6 µl of free water and 2 µl of sample DNA. Afterwards, 8 µl of mineral oil was added on the top of the mixture in each PCR tube.

Reactions were performed according to BioIngenTech instruction: initial denaturation at 94 °C for 2 min, 30 cycles of amplification (denaturation at 94 °C for 30 s, annealing at 57 °C for 30 s, extension at 72 °C for 30 s) and final extension at 72 °C for 5 min.

### Electrophoresis

Separation of amplification products was performed with electrophoresis (90 V, 80 min for *B. burgdorferi* s.l.; 90 V, 60 min for *A. phagocytophilum*; 90 V, 45 min for *Babesia* spp.; 100 V, 45 min for *Rickettsia* spp., *Bartonella* spp. and *C. burnetii*) on 2% agarose gel (Sigma–Aldrich) stained with ethidium bromide (5 µg/ml; Syngene, USA). UV illumination in Gel Logic System 100 (Kodak Imaging System, USA) was used to visualize the amplicons.

For *B. burgdorferi* s.l., positive samples showed amplification products of 120 bp long (fragments of 16S rRNA gene). Additionally, 168-bp long fragments of internal standard were detected in all samples (Moniuszko et al. 2014; Dunaj et al. 2018).

*Anaplasma phagocytophilum* infection was detected in case of presence of the 16S rDNA gene fragments: 932 bp long in PCR-OUT and 546 bp long in PCR-IN. Absence of 932-bp long fragments in PCR-OUT did not exclude a positive result of test (Moniuszko et al. 2014; Dunaj et al. 2018). Positive results for *Babesia* spp. were approximately 420-bp long fragments of the 18S rDNA gene (Moniuszko-Malinowska et al. 2016).

For *Bartonella* spp., *C. burnetii* and *Rickettsia* spp., 140-bp long fragments of internal standard were detected in all samples. Amplification products with the length of 358, 340 and 322 bp were considered positive for *Bartonella* spp., *C. burnetii* and *Rickettsia* spp., respectively.

### Sequencing analysis

#### *Babesia* spp. sequencing

All samples positive for *Babesia* spp. 18S rRNA gene fragment amplicons were sequenced by Macrogen (Amsterdam, The Netherlands), with specific primers used previously for PCR. 5 μl of *Babesia* spp. 18S rDNA amplicons obtained in PCR were combined with 5 μl of each primer (50 mM) and sent to Macrogen where they were sequenced from both sides. The results were later compared with sequences already deposed in the GenBank database using the BLAST server.

#### *Borrelia burgdorferi* s.l. sequencing

5 μl of *B. burgdorferi* s.l. 16S rDNA amplicons obtained in PCR were combined with BIG BOR-F1 (5 μl 50 mM) and BIG BOR-R1 (5 μl 50 mM) and sent to Macrogen. Sequencing was performed using both sides of the DNA strand with primers 16S rDNA BIG BOR-F1 and 16S rDNA BIG BOR-R1 under conditions identical to those used in the original amplification. Afterwards, the sequences were compared with those deposed in the GenBank using the BLAST server.

### Evolutionary analysis by maximum likelihood

The evolutionary history of the various *Babesia* genospecies was inferred by using the Maximum Likelihood method and the Tamura-Nei model (Tamura and Nei 1993). Initial tree(s) for the heuristic search were obtained automatically by applying Neighbor-Join and BioNJ algorithms to a matrix of pairwise distances estimated using the Tamura-Nei model, and then selecting the topology with superior log likelihood value. This analysis involved 34 nucleotide sequences. Codon positions included were 1st + 2nd + 3rd + Noncoding. There were in total 358 positions in the final dataset. Evolutionary analyses were conducted in MEGA X (Kumar et al. 2018).

## Results

In total, 368 *D. reticulatus* were collected in the study area (221 females, 145 males, two nymphs). Overall, 10.3% of *D. reticulatus* ticks were infected (38/368; 21 females, 16 males, one nymph). *Babesia* spp. was detected in 9.2% of the examined ticks (34/368; 20 females, 13 males, one nymph). *Anaplasma phagocytophilum* was confirmed in 0.8% of the ticks (3/368; one female, two males). One male was infected with *B. burgdorferi* s.l. (0.3%; 1/368). *Rickettsia* spp., *Bartonella* spp. and *C. burnetii* were not identified in any of the examined ticks (Table 1). No coinfections were detected.

**Table 1:** Prevalence (%; in parentheses: no. infected/no. examined) of *Borrelia burgdorferi* s.l., *Anaplasma phagocytophilum*, *Babesia* spp., *Rickettsia* spp., *Bartonella* spp., and *Coxiella burnetii* in *Dermacentor reticulatus* ticks collected in Białystok, Poland

Pathogens	*Dermacentor reticulatus* ticks		
	Females	Males	Nymphs
Overall prevalence	9.5 (21/221)	11.0 (16/145)	50 (1/2)
*B. burgdorferi* s.l.	0 (0/221)	0.7 (1/145)	0 (0/2)
*A. phagocytophilum*	0.5 (1/221)	1.4 (2/145)	0 (0/2)
*Babesia* spp.	9.0 (20/221)	8.9 (13/145)	50 (1/2)
*Rickettsia* spp.	0 (0/221)	0 (0/145)	0 (0/2)
*Bartonella* spp.	0 (0/221)	0 (0/145)	0 (0/2)
*C. burnetii*	0 (0/221)	0 (0/145)	0 (0/2)

Sequencing analysis of *Babesia*-positive samples identified 79.4% (27/34; 17 females, nine males, one nymph) of them as *B. canis* with homology ranging from 85.8 to 99.0%. Among the remaining isolates, 8.8% were identified as *B. microti* (3/34; one female, two males) [Accession no. KP055650.1], 5.9% as *Babesia* spp. (2/34; one female, one male) [KX857475.1, KJ956783.1], 2.9% as *B. venatorum* (1/34; one female) [KR003829.1] and 2.9% as *B. vogeli* (1/34; one female) [MT821127.1] (Table S1).

One sample positive for *B. burgdorferi* s.l. was sequenced and showed 98.1% homology with *B. afzelii* [MW301927.1].

### Evolutionary analysis by maximum likelihood

**Fig. 1:**
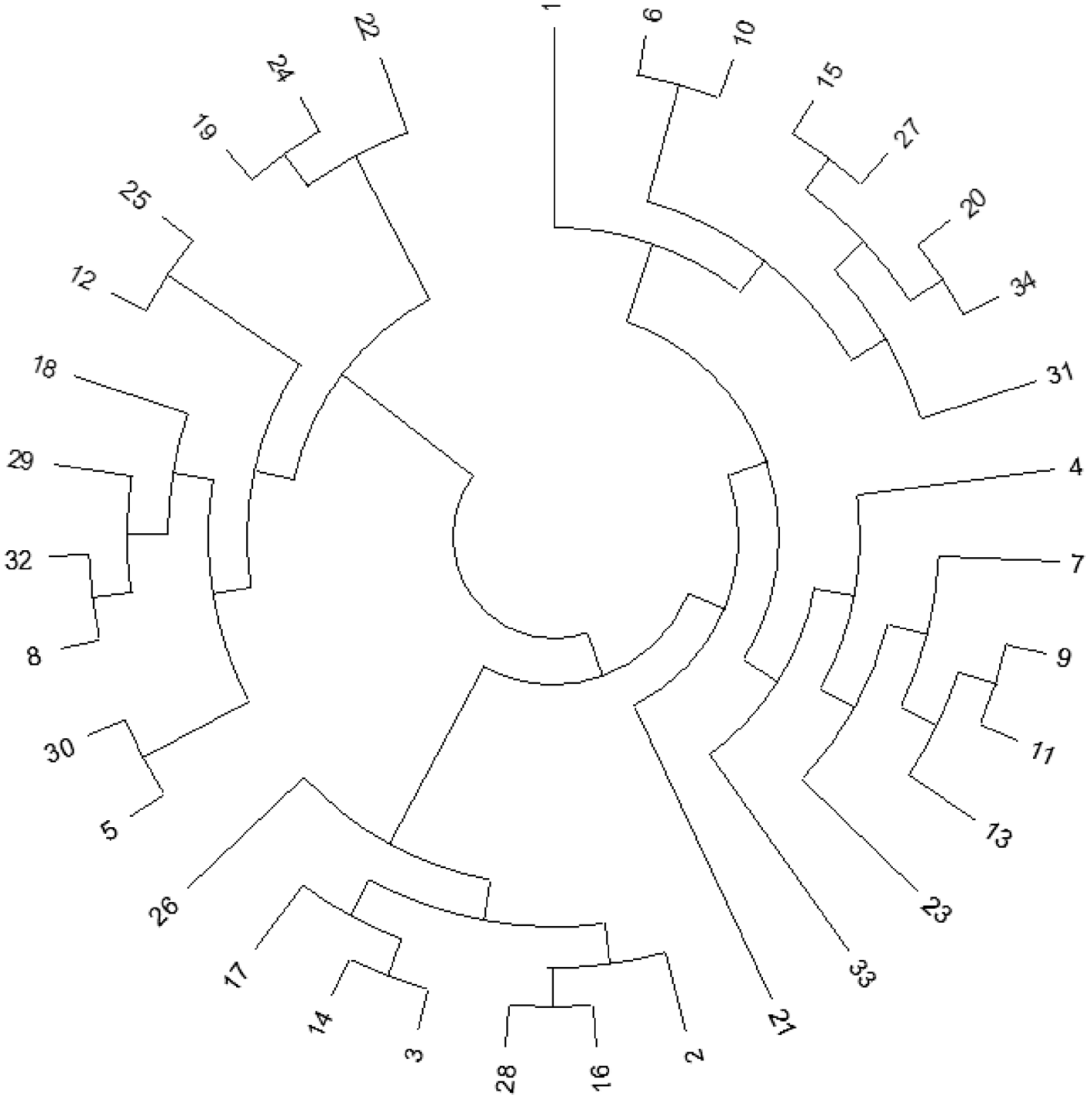
Evolutionary 18S rRNA analysis of the 34 *Babesia* spp. isolates obtained from *Dermacentor reticulatus* ticks within the city of Białystok (see Table S1 for further details), inferred by using the maximum likelihood method and the Tamura-Nei model

The evolutionary history of the various *Babesia* genospecies was inferred by using the Maximum Likelihood method and the Tamura-Nei model. The *Babesia* spp. subpopulation was strongly structured into four genetic clusters, one of which was relatively distant from the others. Interestingly, *B. canis* (21) fell outside the distinguished clusters, which may suggest its primal origin outside from the sampling region. The tree with the highest log likelihood (−12665.52) is shown in Fig. 1.

## Discussion

Although *D. reticulatus* is a commonly found tick species in Europe, literature data regarding their infection rates, especially in urban areas, are much scarcer than such data for *I. ricinus*. To the best of our knowledge, this is the first report on *Babesia* spp., *A. phagocytophilum*, *C. burnetii* and *Rickettsia* spp. prevalence in *D. reticulatus* ticks collected from urban landscapes in Poland.

In this study, the most prevalent pathogen identified in *D. reticulatus* ticks was *Babesia* spp. (9.2%). Studies conducted in rural areas in Poland report noticeably lower prevalence: 4.2% in the Masovian region (central Poland) (Mierzejewska et al. 2015) and 2.5–4.5% in Lublin province (eastern Poland) (Wójcik-Fatla et al. 2012; Zając et al. 2017). Similarly, 5.1% prevalence was reported within the city of Kyiv, Ukraine (Rogovskyy et al. 2018) and in rural landscapes of Serbia (5.7%) (Tomanovic et al. 2013). Radzijevskaja et al. (2018) investigated adult *D. reticulatus* ticks collected from rural areas in Lithuania and Latvia, and found infection rates for *Babesia* spp. of 1.2 and 2.8%, respectively. Studies from Germany (urban areas) and Belarus (rural areas and ticks collected from cattle) reported 0–0.3% *Babesia* spp. infection rate (Silaghi et al. 2012, 2020; Reye et al. 2013).

Sequencing analysis of *Babesia*-positive samples identified the majority of isolates as *B. canis*, of which *D. reticulatus* is a known vector (René-Martellet et al. 2015; Sprong et al. 2019). In relation to the total number of ticks tested, in this study *B. canis* accounted for 6.8% (25/368). Other studies report infection rates ranging from 0.63 to 4.8% (Mierzejewska et al. 2015; Zając et al. 2017; Radzijevskaja et al. 2018; Rogovskyy et al. 2018). Noticeably higher prevalence was reported by Dzięgiel et al. (2014) (21.3%) and Tomanovic et al. (2013) (20.8%).

*Babesia microti* is considered to be the most common causative agent for human babesiosis (Vannier et al. 2008). Its primary tick vectors are *Ixodes scapularis* and *I. ricinus* (Vannier et al. 2008; Wójcik-Fatla et al. 2012). To date, limited data are available regarding the potential role of *D. reticulatus* as a vector for *B. microti*. Research conducted by Wójcik-Fatla et al. (2012) confirmed the presence of *B. microti* in 4.5% of tested ticks. As stressed by the authors, this was the first report on *B. microti* presence in adult *D. reticulatus*. In the present study, *B. microti* accounted for 0.8% of examined ticks and was also found only in adults. In other studies detected prevalence rate was 0.04–4.0% (Mierzejewska et al. 2015; Opalińska et al. 2016; Zając et al. 2017). To the best of our knowledge, this is the first report of *B. microti* presence in questing adult *D. reticulatus* in urban surroundings. Although this tick rarely feeds on humans, presence of *B. microti* within the city is of epidemiological importance.

In this study, one adult tick was infected with *B. venatorum*, which is another causative agent for human babesiosis (Hildebrandt et al. 2013). To date, only two other studies, from Lithuania and Russia, reported the presence of this pathogen in *D. reticulatus*. In both, *B. venatorum* was detected in a single tick (Livanova et al. 2018; Radzijevskaja et al. 2018).

*Babesia vogeli* is one of the causative agents of canine babesiosis. In Europe, this pathogen is commonly found in the Mediterranean area and transmitted by *Rhipicephalus sanguineus*, which is the predominant tick species there (René-Martellet et al. 2015). To date, no other research confirmed *B. vogeli* in *D. reticulatus*.

In the current study, 0.8% of *D. reticulatus* ticks was infected with *A. phagocytophilum*. In Poland, similarly low prevalence was reported in rural areas in the eastern region (1.1%) (Zając et al. 2017), whereas in western provinces *A. phagocytophilum* was not detected in any of the tested *D. reticulatus* ticks (Opalińska et al. 2016). Prevalence of *A. phagocytophilum* was 0–1% in the city of Kyiv, Ukraine (Didyk et al. 2017; Rogovskyy et al. 2018), 0% in Germany (Richter et al. 2013) and 1.9% in rural areas of Serbia (Tomanovic et al. 2013).

In this study, the presence of *B. afzelii* was confirmed in only one *D. reticulatus* tick (0.3%). Low prevalence has been reported by several other studies in Poland: 0.09% in Masovian Voivodeship (rural areas) (Mierzejewska et al. 2015) and 0.6–1.6% in Lubelskie Voivodeship (rural areas) (Dzięgiel et al. 2014; Zając et al. 2017). Low prevalence was also reported for *B. burgdorferi* s.l. infection in *D. reticulatus* ticks, such as 2.0% in Wroclaw Agglomeration (urban areas) (Król et al. 2015), as well as in other European countries (studies in rural landscapes): 1.8% in Belarus (Reye et al. 2013) and 0% in Serbia (Tomanovic et al. 2013) and Germany (Richter et al. 2013). A possible explanation for such low prevalence may be found in research conducted by Johns et al. (2001) who observed *D. variabilis* to be highly immunocompetent against *Borrelia* spirochetes. Rudolf et al. (2003) demonstrated the inhibition of *Borrelia* bacteria growth by extracts from the midguts of *D. reticulatus* (in vitro). Based on these findings, *Dermacentor* ticks appear ineffective vectors for *B. burgdorferi* s.l.

No *D. reticulatus* ticks examined in this study tested positive for presence of *C. burnetii*. Similar results were obtained by Tylewska-Wierzbanowska et al. (1996). Consistent values have been reported in rural areas in Germany and Belarus (Pluta et al. 2010; Reye et al. 2013), whereas in Slovakia studies show 0–2.1% prevalence (Smetanová et al. 2006; Špitalská et al. 2018). In Serbia, Tomanovic et al. (2013) obtained 3.7% *C. burnetii* infection rate. In comparison, Bonnet et al. (2013) confirmed the presence of *C. burnetii* in 16% of *D. reticulatus* ticks collected in various rural locations in France.

*Bartonella* spp*.* was not detected in any of the *D. reticulatus* ticks tested in this study. This pathogen was found to be present in one *D. reticulatus* (0.5% of all examined ticks) collected from vegetation in an urban park in Warsaw, Poland (Podsiadly et al. 2009). Such low prevalence was also reported in studies from rural areas in Belarus (0.6%) (Reye et al. 2013) and Serbia (0%) (Tomanovic et al. 2013), as well as in the city of Kyiv, Ukraine (1.0%) (Rogovskyy et al. 2018).

No *D. reticulatus* ticks tested positive for the presence of *Rickettsia* spp. This is especially surprising, giving that other research conducted in Podlaskie voivodeship reported 40.7–56.7% prevalence, even though those studies were conducted in rural landscapes (Stańczak 2006; Chmielewski et al. 2009; Stańczak et al. 2018). High *Rickettsia* spp. infection rates (41.8–53.0%) were reported from various regions of Poland (Wójcik-Fatla et al. 2013; Mierzejewska et al. 2015; Zając et al. 2017). Interestingly, prevalence reported in other European countries is not as high: 10.1–35.7% in Ukraine (urban areas) (Didyk et al. 2017; Rogovskyy et al. 2018), 14% in The Netherlands (rural areas) (Nijhof et al. 2007) and 21.4% in Germany (rural areas) (Dautel et al. 2006).

In conclusion, results obtained in this study provide valuable information about prevalence of tick-borne pathogens in *D. reticulatus* ticks. Specimens were infected with at least three pathogens: *B. burgdorferi* s.l., *A. phagocytophilum* and *Babesia* spp. Additionally, this research provided the first identification of *B. vogeli* in *D. reticulatus* ticks. Therefore, further investigation is necessary in order to estimate the risk of human and animal infection.

## Supplementary Information

Below is the link to the electronic supplementary material.

Supplementary file1 (DOCX 20 kb)

## Data Availability

All data generated or analyzed during this study are included in this published article.
