# Correction to: Detection of *Borrelia burgdorferi* s.l., *Anaplasma phagocytophilum* and *Babesia* spp. in *Dermacentor reticulatus* ticks found within the city of Białystok, Poland—first data

**DOI:** 10.1007/s10493-022-00712-z

**Published:** 2022-04-22

**Authors:** Anna Grochowska, Justyna Dunaj, Sławomir Pancewicz, Piotr Czupryna, Piotr Majewski, Mulugeta Wondim, Elżbieta Tryniszewska, Anna Moniuszko-Malinowska

**Affiliations:** 1 Department of Infectious Diseases and Neuroinfections, Medical University of Białystok, Żurawia 14, 15-540 Białystok, Poland; 2 Department of Microbiological Diagnostics and Infectious Immunology, Medical University of Białystok, Waszyngtona 15A, 15-269 Białystok, Poland

## Correction to: Experimental and Applied Acarology (2021) 85:63–73 10.1007/s10493-021-00655-x

In the original publication, an incorrect GenBank Accession number was displayed in the Results section at the bottom of page 67. The sequence number KR003829.1 should have read: KR003828.1. The numbers in the supplementary Table S1 are correct.

