# Supplementary material for: Detection of Borrelia burgdorferi s.l., Anaplasma phagocytophilum and Babesia spp. in Dermacentor reticulatus ticks found within the city of Białystok, Poland—first data
Source: Exp Appl Acarol. 2021 Sep 15;85(1):63–73. doi: 10.1007/s10493-021-00655-x (PMC8486709; doi:10.1007/s10493-021-00655-x)
Supplement: Supplementary file 1 — Supplementary file1 (DOCX 20 kb) [file 10493_2021_655_MOESM1_ESM.docx]

**Table S1** Results of *Babesia* 18S rRNA sequencing.

|  | **Genospecies** | **Homology (%)** | **GenBank Accession** | **Lenght (no. bp)** |
| --- | --- | --- | --- | --- |
| 1 | *B. canis* | 95.38 | MN704759.1 | 483 |
| 2 | *B. canis* | 88.62 | MN173223.1 | 346 |
| 3 | *B. canis* | 94.44 | MT346582.1 | 520 |
| 4 | *B. canis* | 87.67 | MN708319.1 | 516 |
| 5 | *B. canis* | 93.73 | MF797820.1 | 451 |
| 6 | *B. venatorum* | 91.83 | KR003828.1 | 425 |
| 7 | *B. canis* | 94.52 | MN704759.1 | 532 |
| 8 | *B. canis* | 89.50 | MN173223.1 | 429 |
| 9 | *B. canis* | 96.83 | MN704759.1 | 569 |
| 10 | *B. canis* | 95.89 | MN078319.1 | 512 |
| 11 | *Babesia* sp. | 95.83 | KX857475.1 | 427 |
| 12 | *B. vogeli* | 94.87 | MT821127.1 | 427 |
| 13 | *B. canis* | 85.79 | MF797820.1 | 374 |
| 14 | *B. canis* | 91.05 | KU821654.1 | 435 |
| 15 | *B. canis* | 98.98 | MN078319.1 | 492 |
| 16 | *B. canis* | 86.60 | Mn173223.1 | 318 |
| 17 | *B. canis* | 89.16 | MN540622.1 | 351 |
| 18 | *B. canis* | 95.99 | MN704759.1 | 473 |
| 19 | *B. microti* | 93.69 | KP055650.1 | 449 |
| 20 | *B. canis* | 95.41 | KT844881.1 | 486 |
| 21 | *B. canis* | 95.17 | KP835549.1 | 459 |
| 22 | *B. canis* | 94.06 | MN173223.1 | 486 |
| 23 | *B. canis* | 95 | FJ913767.1 | 509 |
| 24 | *B. microti* | 95.93 | KP055650.1 | 505 |
| 25 | *B. microti* | 95.83 | KP055650.1 | 427 |
| 26 | *B. canis* | 90.40 | MN540626.1 | 420 |
| 27 | *B. canis* | 89.32 | MN173223.1 | 420 |
| 28 | *B. canis* | 98.98 | KT844899.1 | 525 |
| 29 | *Babesia* sp. | 89.62 | KJ956783.1 | 366 |
| 30 | *B. canis* | 90.47 | MN173222.1 | 316 |
| 31 | *B. canis* | 86.20 | MN078319.1 | 318 |
| 32 | *B. canis* | 91.03 | AY259123.1 | 403 |
| 33 | *B. canis* | 94.43 | MN078320.1 | 468 |
| 34 | *B. canis* | 95.17 | MN078319.1 | 463 |
